# Late-Onset Axial Spondyloarthritis in an Elderly Patient: A Case Report

**DOI:** 10.7759/cureus.111089

**Published:** 2026-06-18

**Authors:** Phengann Kao, Sakseth Sam, Meyyien Eang, Leangsing Iv, Mala Ngy

**Affiliations:** 1 Medicine A Department, Calmette Hospital, Phnom Penh, KHM; 2 Medicine A Department, University of Health Science, Phnom Penh, KHM; 3 Radiology Department, Calmette Hospital, Phnom Penh, KHM

**Keywords:** asas classification criteria, axial spondyloarthritis, elderly patient, late onset, rare case

## Abstract

Spondyloarthritis is a group of chronic inflammatory rheumatic diseases characterized by predominant involvement of the axial skeleton (spine and sacroiliac joints) and/or peripheral joints, along with inflammation at sites of tendon and ligament insertion (enthesitis). The disease commonly affects young adults. However, late-onset spondyloarthritis in an elderly patient is a very rare condition, but it can occur. We report a 57-year-old woman admitted to the ​Medicine A Department of Calmette Hospital in April 2026 for two years of chronic inflammatory pain in the left buttock associated with peripheral joint pain. The symptoms meet the full criteria for axial spondyloarthritis, except for the age of the patient.

Spondyloarthritis is a chronic inflammatory rheumatic disease that commonly affects young adults, with onset usually before the age of 45 years. Our case highlights the need to re-evaluate the role of the age-at-onset criterion in the Assessment of SpondyloArthritis international Society (ASAS) classification system. While the current criteria perform well in younger populations, rare cases of late-onset spondyloarthritis suggest that some patients may not be adequately captured. Larger epidemiological and validation studies are needed before any modification of the classification criteria can be recommended.

## Introduction

Spondyloarthritis is a group of chronic inflammatory rheumatic diseases characterized by predominant involvement of the axial skeleton (spine and sacroiliac joints) and/or peripheral joints, along with inflammation at sites of tendon and ligament insertion (enthesitis) [1]. The disease commonly affects young adults, with onset usually before the age of 45 years, and shows a slight male predominance, particularly in axial forms [2]. The clinical manifestation of spondyloarthritis presents with inflammatory back pain, characterized by insidious onset, morning stiffness, and improvement with exercise [3]. Other key features include peripheral arthritis (often asymmetric), enthesitis, and dactylitis, along with extra-articular manifestations such as uveitis, psoriasis, and inflammatory bowel disease [4]. However, late-onset spondyloarthritis in an elderly patient is a very rare condition, but it can occur [5].

## Case presentation

A 57-year-old woman was admitted to the Medicine A Department of Calmette Hospital in April 2026 with a two-year history of inflammatory pain involving the left buttock, both shoulders, and knees. The pain had an insidious onset and progressively worsened over time. She reported associated morning stiffness lasting more than 30 minutes, with noticeable improvement following physical activity. Despite the chronicity and progression of her symptoms, she had not undergone any previous rheumatologic evaluation but took auto medication with painkillers and non-steroidal anti-inflammatory drugs without any improvement.

She denied any history of trauma, fever, peripheral joint swelling, psoriasis, inflammatory bowel disease, uveitis, or recent genitourinary or gastrointestinal infection. No other extra-articular manifestations were reported. Her past medical history was significant for type 2 diabetes mellitus and hypertension. She denied smoking and alcohol consumption.

On physical examination, there was no tenderness or limitation of movement in either shoulder or knee. Tenderness was noted over the left sacroiliac region. Sacroiliac joint stress maneuvers were painful, including a positive FABER (Patrick's) test on the left side. Lumbar spine mobility was preserved. No peripheral joint swelling, deformity, or tenderness was observed. There was no evidence of enthesitis or dactylitis. Neurological examination was unremarkable, with no motor or sensory deficits. The remainder of the physical examination was normal.

Laboratory investigations (Table 1) revealed a white blood cell count of 6.6 × 10⁹/L (reference range: 4-9 × 10⁹/L), hemoglobin of 117 g/L (reference range: 130-170 g/L), and platelet count of 270 × 10⁹/L (reference range: 150-450 × 10⁹/L). Inflammatory markers were elevated, with a C-reactive protein (CRP) level of 15 mg/L (reference range: <5 mg/L) and an erythrocyte sedimentation rate (ESR) of 150 mm/h. Serum creatinine was 89 μmol/L (reference range: 62-115 μmol/L), blood urea nitrogen was 0.61 g/L (reference range: 0.18-0.55 g/L), and uric acid was 45 mg/L (reference range: 35-72 mg/L). HLA-B27 test was not requested because it was not available at our hospital. Rheumatoid factor and anti-cyclic citrullinated peptide antibodies were negative.

**Table 1:** Laboratory investigations at admission

Parameter	Result	Reference range	Interpretation
White blood cell count	6.6 × 10⁹/L	4-9 × 10⁹/L	Within normal limits
Hemoglobin	117 g/L	130-170 g/L	Mild anemia
Platelet count	270 × 10⁹/L	150-450 × 10⁹/L	Within normal limits
C-reactive protein	15 mg/L	<5 mg/L	Elevated, indicating active inflammation
Erythrocyte sedimentation rate	150 mm/h	<20 mm/h	Markedly elevated, consistent with significant inflammatory activity
Serum creatinine	89 μmol/L	62-115 μmol/L	Normal renal function
Blood urea nitrogen	0.61 g/L	0.18-0.55 g/L	Mildly elevated
Uric acid	45 mg/L	35-72 mg/L	Within normal limits
Rheumatoid factor	Negative	Negative	Negative serological markers for rheumatoid arthritis
Anti-cyclic citrullinated peptide antibodies	Negative	Negative	Negative serological markers for rheumatoid arthritis
HLA-B27	Not performed	—	HLA-B27 testing was unavailable at our institution

Plain radiographs of both shoulders, both knees, and the sacroiliac joint showed no abnormalities. Magnetic resonance imaging (MRI) of the sacroiliac joints demonstrated active inflammatory changes with bone marrow edema, predominantly affecting the left sacroiliac joint (Figure 1). These findings were highly suggestive of active sacroiliitis in the setting of axial spondyloarthritis. According to the Assessment of SpondyloArthritis International Society (ASAS) classification criteria, the presence of sacroiliitis on MRI, together with two additional spondyloarthritis features (inflammatory buttock pain and elevated inflammatory markers), supports the classification of this patient as having axial spondyloarthritis, irrespective of age [6].

**Figure 1:**
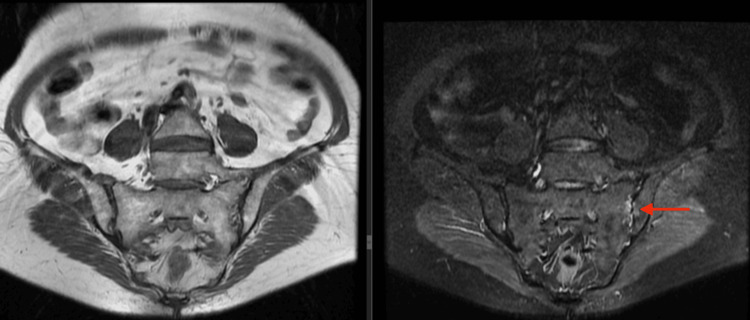
MRI of the sacroiliac joints in STIR sequence showing left-side sacroiliitis characterized by increased signal intensity, depicting bone marrow edema in the left side of sacroiliac joints. STIR, short tau inversion recovery.

She underwent treatment with a Janus-activated kinase inhibitor, which is the most commonly used targeted therapy in our country for the second-line treatment of inflammatory rheumatic disease. At the one-month follow-up, she reported no more pain in the affected joints. There was no tenderness on sacroiliac joint examination. Her inflammatory markers had decreased, with a CRP level of 4 mg/L (previously 15 mg/L).

## Discussion

Spondyloarthritis is a chronic inflammatory rheumatic disease characterized by involvement of the axial skeleton, peripheral joints, and entheses. Disease onset typically occurs before the age of 45 years; therefore, the diagnosis may be overlooked in older adults presenting with musculoskeletal symptoms. Late-onset spondyloarthritis, usually defined as disease onset after the age of 50 years, represents a less common but increasingly recognized clinical entity.

Our patient presented at the age of 57 years with a two-year history of inflammatory buttock pain and prolonged morning stiffness, associated with inflammatory polyarthralgia involving both shoulders and knees. Although shoulder and knee pain were painful, physical examination did not reveal objective peripheral arthritis. The presence of inflammatory buttock pain prompted further investigation, leading to an MRI of the sacroiliac joints, which demonstrated active sacroiliitis. These findings fulfilled the clinical and imaging features suggestive of axial spondyloarthritis.

In a previous case report, Voulgari et al. [7] have also reported a 70-year-old woman who presented with fever of unknown origin, back pain, malaise, weakness, and high acute phase reactants, without any other clinical or laboratory findings. After an intensive and extensive investigation to exclude several diseases and disorders, they concluded the diagnosis of late-onset axial spondyloarthritis. The patient was treated successfully with adalimumab.

Chung et al. [8] reported a case of a 66-year-old man who presented with pain and swelling of the left knee for four months, followed by progressive involvement of the right knee, right ankle, and the first to third toes of the left foot. He also experienced fever and weight loss of 4 kg over a three-month period. His medical history was notable for bilateral total hip replacement surgeries performed years earlier because of unexplained hip arthritis.

Physical examination revealed swelling and tenderness of both knees, swelling of the right ankle, and arthritis involving the left first to third toes. He did not report inflammatory back pain, and the FABER test was negative. Laboratory investigations demonstrated anemia (hemoglobin: 10.2 g/dL), leukocytosis (12,790/mm³), thrombocytosis (670,000/mm³), an ESR of 97 mm/h, and a CRP level of 102.7 mg/L. Rheumatoid factor and antinuclear antibody tests were negative, while HLA-B27 was positive. The patient was diagnosed with late-onset peripheral spondyloarthritis and was successfully treated with disease-modifying antirheumatic drugs.

Olivieri et al. [9] reported that approximately 3.5%-6% of patients with axial spondyloarthritis experienced disease onset after the age of 45 years. However, the ASAS classification criteria are restricted to patients with chronic back pain onset before the age of 45 years. This age restriction may represent a limitation of the criteria, as it could lead to the underrecognition of late-onset axial spondyloarthritis. 

## Conclusions

Spondyloarthritis is a chronic inflammatory rheumatic disease that commonly affects young adults, with onset usually before the age of 45 years. Our case highlights the need to re-evaluate the role of the age-at-onset criterion in the ASAS classification system. While the current criteria perform well in younger populations, rare cases of late-onset spondyloarthritis suggest that some patients may not be adequately captured. Larger epidemiological and validation studies are needed before any modification of the classification criteria can be recommended.
